# Adaptations and modifications to *the 15-method* in Danish general practice classified using the framework for reporting adaptations and modifications to evidence-based interventions (FRAME)

**DOI:** 10.1186/s13722-025-00613-7

**Published:** 2025-10-27

**Authors:** Peter Næsborg Schøler, Per Nilsen, Sanne Rasmussen, Jens Søndergaard, Anette Søgaard Nielsen

**Affiliations:** 1 Department of Clinical Research, Unit for Clinical Alcohol Research, Research Unit of Psychiatry, University of Southern Denmark, J.B. Winsløwsvej 18, ent. 220B, First Floor, Odense C, DK-5000 Denmark; 2 Department of Public Health, Research Unit of General Practice, University of Southern Denmark, Odense, Denmark; 3 Department of Psychiatry Odense, Mental Health Services Region of Southern Denmark, Odense, Denmark; 4 Department of Health, Medicine and Caring Sciences, Linköping University, Linköping, Sweden; 5 BRIDGE, Brain Research - Inter Disciplinary Guided Excellence, University of Southern Denmark, Odense, Denmark

**Keywords:** Screening and brief intervention, Framework for reporting adaptations and modifications to evidence-based interventions, Alcohol intervention, Implementation science, Primary care

## Abstract

**Background:**

Unhealthy alcohol use is a major global public health problem, yet alcohol problems often remain unaddressed in primary care. The 15-method, developed in Sweden, offers a flexible, patient-centered approach to alcohol-related issues using opportunistic screening and stepped-care treatment in the same setting. The present study describes adaptations and modifications made to the 15-method by Danish healthcare professionals during a randomized controlled trial testing the 15-method’s effectiveness in Danish general practice.

**Methods:**

Data for the present qualitative study were individual and group interviews with 28 healthcare professionals from 12 Danish general practices enrolled in the Identification and Treatment of Alcohol Problems in Primary Care (iTAPP) study, a stepped-wedge cluster randomized controlled trial. Interviews focused on innovation and implementation process determinants from the Consolidated Framework for Implementation Science (CFIR). By means of the Framework for Reporting Adaptations and Modifications to Evidence-based interventions (FRAME), data were analyzed to identify modifications to the 15-method when implemented in daily use.

**Results:**

Four distinct modifications were identified: a loosening of the method’s structure, condensed use of the method’s materials, modification of screening procedures, and integration into existing clinical procedures. Half of the changes were unplanned, and half were fidelity-consistent. Three of four changes occurred on practice level and were made by general practitioners. The most common goal of the changes was to increase patient reach and engagement. More training and structured follow-up were also identified as important to increase healthcare professionals’ confidence in delivering the 15-method.

**Conclusion:**

General practitioners and nurses generally find the 15-method suitable for their work in Danish general practice, and the method can be readily implemented with minor adaptations. More training and implementation planning may promote higher utilization and more consistent use, ultimately increasing the method’s sustainability and effectiveness.

**Supplementary Information:**

The online version contains supplementary material available at 10.1186/s13722-025-00613-7.

## Introduction

Interventions targeting alcohol-related problems and unhealthy alcohol use present significant potential for public health benefits in both primary and secondary healthcare settings [1–3]. Although screening and brief intervention (SBI) strategies have proven effective in reducing unhealthy alcohol use [4], their implementation in routine healthcare is often hampered by reoccurring barriers such as fear of damaging the patient-provider relationship and lack of resources among healthcare professionals (HCPs) [5, 6], along with self-stigmatization and beliefs in self-management of the problem among patients [7, 8]. The implementation challenge has been likened to fitting a square peg into a round hole, as the conditions under which SBI research is typically conducted differ significantly from those found in real-world practice [9, 10].

In response to the challenge of implementing SBI, the “15-method” has been developed as a screening and brief intervention tool. The 15-method is described in detail in the Methods section below. Briefly, the 15-method is designed to support healthcare professionals in addressing and treating alcohol-related problems through a patient-centered, stepped-care approach. Initially developed and tested in Swedish primary care, the 15-method demonstrated promising results [11, 12]. A subsequent feasibility study in Danish general practice showed that both HCPs and patients found the method helpful and usable, although certain areas of improvement were noted [13]. Consequently, the 15-method was adapted to align better with Danish cultural norms and the context of Danish general practice, using a participatory design approach [14].

To assess the effectiveness of the Danish version of the 15-method, the Identification and Treatment of Alcohol Problems in Primary Care (iTAPP) Study was initiated [15]. The iTAPP study involves 21 Danish general practices which implemented the 15-method into their daily work. The iTAPP study follows the Medical Research Council’s framework for developing and evaluating complex interventions [16] and includes an implementation evaluation informed by the Consolidated Framework for Implementation Research (CFIR), which identifies contextual and intervention-specific factors that impact implementation success including an Implementation Process Domain focusing on the activities and strategies used to implement the intervention [17].

Adaptations and modifications to interventions are common in clinical trials and in real-world implementation, reflecting changes made to better align with specific needs, resources and context of those both delivering and receiving the intervention [18]. Adaptations are generally defined as the intentional changes to an intervention with the goal of enhancing its effectiveness or relevance in a specific context, such as translating materials and incorporating culturally relevant examples. Where adaptations strive to preserve core elements of the intervention and maintain fidelity, modifications are changes made in response to situational constraints or challenges that may compromise the theoretical and practical integrity of the intervention [18, 19], such as reducing the number of consultations in a specified treatment protocol.

Tracking changes, i.e., adaptations and modifications, to interventions is crucial, as they potentially enhance intervention acceptability, but may also introduce fidelity-inconsistent changes that negatively affect both intended intervention outcomes and the assessments of intervention effectiveness [16, 20–22]. Therefore, identifying and understanding adaptations or modifications made in real-world settings is key to accurately evaluating trial outcomes, balancing fidelity with adaptability, and optimizing intervention uptake to ultimately enhance intervention effectiveness [22–24].

In light of this, the present study seeks to clarify whether and how the 15-method was adapted and modified in the iTAPP study, utilizing the Framework for Reporting Adaptations and Modifications to Evidence-based interventions (FRAME) [25]. Specifically, this study aimed to identify and characterize adaptations and modifications to the 15-method intervention made by healthcare providers during implementation in Danish general practice settings.

## Methods and materials

### Design

**Fig. 1:**
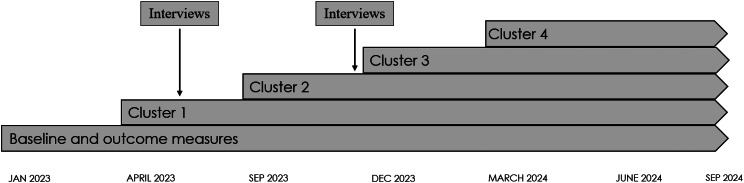
Timeline and interview timepoints for evaluating adaptations and modifications to the 15-metod during implementation in the iTAPP study. Total of 28 healthcare professionals across 12 general practices in The Region of Southern Denmark. Notes: iTAPP, Identification and Treatment of Alcohol Problems in Primary Care (iTAPP) study. Stepped-wedge cluster randomized design. Baseline and outcome measures refer to measures in the iTAPP study. Each cluster includes 4–6 general practices. Interviews (*n* = 17) consisted of 6 individual and 11 group interviews

The present study was based on data from individual interviews and group interviews. The interviews were conducted in two rounds during the iTAPP study (Fig. 1), which is a stepped-wedge cluster randomized control trial [26]. In the iTAPP study, four clusters of general practices were enrolled as active practices, i.e. implementing the 15-method, in three-month intervals following a three-month baseline period [15]. The present study adheres to the Standards for Reporting Qualitative Research (SRQR) [27].

### Setting

The participating general practices are situated in the Region of Southern Denmark, which comprises 345 practices [28] each with an average of 2.3 general practitioners (GPs). In this region, the typical GP manages 1,541 listed patients, and 99% of the Danish population is registered with a GP [29, 30]. The GP functions as the primary provider and a gatekeeper to the secondary healthcare system and medical treatment and consultations incur no costs for patients [31]. The practices involved in the iTAPP study encompass both urban and rural settings and include solo practitioners as well as larger practices with several GPs, nurses, and other staff groups e.g. medical students and assistants [15, 30].

### Intervention

The 15-method is a three-step intervention designed to help HCPs address alcohol-related issues in patients through a structured approach.

Step one (initial screening and brief advice): during routine visits, HCPs conduct opportunistic screening and offer brief advice. HCPs may use screening tools like the Alcohol Use Disorder Identification Test (AUDIT) [32] and biomarkers to screen for alcohol issues if relevant symptoms are present [4, 33]. If alcohol is found to be pertinent to the patient’s condition, the HCPs provide brief advice during this same consultation and suggests scheduling a dedicated follow-up (step two). Between step one and two, patients may complete the AUDIT questionnaire if not already administered to facilitate continuity and ensure comprehensive assessment.

Step two (personalized feedback and check-up): In this second consultation, HCPs provide personalized feedback based on information obtained during step one and between step one and two (e.g., AUDIT results, biomarkers). Tools like the one-week Timeline Follow-Back [34], the Short Alcohol Dependence Data Questionnaire [35], and the International Classification of Diseases 10th revision (ICD-10) criteria for alcohol dependence [36] may be used for further assessment, alongside screening for other substance use if needed, e.g. benzodiazepines and opioids during this step. The HCP then encourage patients to consider the impact of their drinking on health and explore motivation for behavioral change.

Step three (Treatment sessions with planned follow-up): This step comprises up to three treatment sessions, premised on Motivational Interviewing (MI) [37], Cognitive Behavioral Therapy, and Guided Self-Change [38] incorporating strategies like an alcohol diary, goal setting, and self-monitoring exercises. Treatment sessions utilize MI principles, in which Danish GPs receive training during their specialist training. Homework assignments incorporate straightforward CBT-based exercises that require no formal CBT training for HCPs to administer. Collectively, these treatment sessions constitute a guided self-change approach. Treatment intensity and goals are established through shared decision-making [39], with pharmacological treatments options available per national guidelines (Disulfiram, Acamprosate, Nalmefene, or Naltrexone). Step three concludes with collaborative planning between HCP and patient for either a follow-up consultation or referral to specialized treatment, based on the patient’s needs.

HCPs received training in the 15-method and in recognizing and addressing alcohol-related symptoms using an MI approach through academic detailing [40, 41]. Academic detailing, also referred to as educational outreach visits, comprises structured, evidence-based educational interventions delivered through planned visits to healthcare settings to facilitate knowledge translation and clinical practice improvement. Details on the 15-method, its materials, and implementation in the iTAPP study are provided elsewhere [7, 12, 15, 42].

### Recruitment and data collection

Interview participants were recruited through their participation in the iTAPP study as part of the 15-method training sessions. HCPs from cluster one and two were included with representation of both GPs and nurses to ensure data from both managers and staff. Interviews were conducted from May to December 2023 in two rounds (Fig. 1) and included 28 HCPs from twelve of the twenty-one general practices participating in the iTAPP. The interviews (*n* = 17) were 30–60 min and consisted of in-person and video interviews, with 6 individual and 11 group interviews [43, 44]. The first round of interviews held six practices and 12 HCPs, round two was conducted in 11 practices with 25 HCPs including repeat interviews with a subset of participants from round one (Table 1). The participants were interviewed in their practices during working hours and compensated for their time equivalent to their hourly rate. The interviews were semi-structured and guided by the CFIR interview guide available from www.cfirguide.org. We designed the interview guide to have lead questions and follow-up and probe questions within each topic. We used follow-up questions as needed to provide details on a topic, while we omitted questions with information evident prior to the interviews (e.g. role, title). As the FRAME does not provide an explicit interview guide, we compared questions in the FRAME reporting tool to the CFIR interview guide to ensure we covered all relevant aspects related to intervention changes. Supplementary File 1 features the interview guide with a brief description of lead and follow-up questions. For the present study we focused on changes made to the intervention and the CFIR domains Innovation, Inner Setting, and Implementation Process. While this study focuses specifically on these three domains, questions related to the domains “Individual” and “Outer Setting” were included in the interview guide as part of a broader data collection strategy to address multiple research questions across related studies [45]. The first domain, Innovation, includes constructs related to the innovation’s adaptability, trialability, complexity, design, and cost. The Inner Setting domain focuses on the physical infrastructure, work infrastructure, communication, culture, and available resources and materials. The Implementation Process domain focuses on the adaptation process as to the degree to which participants modified and adapted the intervention and/or the inner setting to improve the fit and integration of the intervention into their work [17].

**Table 1:** Overview of interviewed healthcare professionals (*N* = 28)

Practice number	Cluster number in the iTAPP study	Number of interviews (*N* = 17)	Number of participating HCP in interview round one (*n* = 12)	Number of participating HCP in interview round two (*n* = 25)	Type of interview
1	1	2	1 GP	1 nurse	Individual. Video.
2	2	1	-	4 GPs	Group interview. Video.
3	1	2	1 GP	1 GP (same)	Individual. First in person, second via video.
4	1	1	1 nurse	(discontinued)	Individual. Video.
5	1	2	2 nurses	2 nurses (same), 1 GP	Group interview. Video.
6	1	2	1 GP, 2 nurses	2 nurses (same)	Group interview. First in person, second via video.
7	1	2	2 GPs, 2 nurses	2 GPs, 2 nurses (same)	Group interview. In person.
8	2	1	-	1 GP, 2 nurses	Group interview. In person.
9	2	1	-	1 GP, 1 nurse	Group interview. In person.
10	2	1	-	1 GP	Individual. In person.
11	2	1	-	2 GPs	Group interview. Video.
12	2	1	-	2 GPs	Group interview. Video.

Notes: iTAPP, Identification and Treatment of Alcohol Problems in Primary Care, GP, General practitioner. HCP, Healthcare professional

Two research assistants transcribed the interviews verbatim.

### Data storage

Data were stored on secure serves hosted by the Region of Southern Denmark at Odense Patient data Explorative Network (OPEN) [46] in compliance with the European General Data Protection Regulations.

### Analyses

Our analyses were guided by The Consolidated Framework for Implementation Science (CFIR) [17, 47] and The Framework for Reporting Adaptations and Modifications to Evidence-based interventions (FRAME) [18, 20]. Details on both frameworks are provided below. We analyzed data using directed content analysis [48, 49] using predetermined structure and codes from the CFIR and FRAME. We added codes for each FRAME element to an Nvivo 12 template pre-filled with CFIR determinants (codes) [50]. We then coded data according to the CFIR and FRAME codebooks as provided by the Fidelity, Adaptation, Sustainability, and Training (FAST) Lab at Standford University [51] and by the CFIR research team’s website (www.cfirguide.org) [50]. Additionally, we created a case-memo for each practice with reflections and notes for group discussion.

Data on specific adaptations and modifications were coded to the FRAME and then transferred to a fillable FRAME spreadsheet [51]. Implementation determinants coded to the CFIR were cross-referenced with FRAME codes and the spreadsheet. By combining these two frameworks, we aimed to gain deeper insight into the timing, nature, and reasons behind the identified adaptations and modifications in relations to the ongoing implementation process. While the FRAME focuses on the adaptations and modification to the intervention, and not their derivative effects or the surrounding implementation process [52], the CFIR focuses on implementation determinants both related to the intervention and its deliverers, but also the surrounding implementation process and contexts [17]. The CFIR provides insights into the perceived adaptability of the intervention, contextual factors affecting compatibility, and participants’ reflections on their use of the intervention. Conversely, the FRAME classifies changes according to their nature, level of planning, and their relationship to fidelity in an easily accessible format. By combining the two frameworks, we were able to succinctly classify changes to the 15-method and provide insights into the respondents’ considerations and reasoning behind these changes within their ongoing implementation efforts and work contexts. Notably, we assessed all CFIR constructs from the perspective of the HCPs, including the Implementation Process domain and restricted analysis to CFIR domains “Intervention”, “Inner Setting” and “Implementation Process”, with analysis of data from the Individuals Domain and Outer Setting reported elsewhere [45].

PNS conducted the initial coding and discussed findings with the research team. Re-coded materials were discussed again until consensus. The overall analytic framework and process was overseen by a qualitative research expert unaffiliated with the research group.

### Framework for analyzing adaptations and modifications to the intervention: FRAME

The Framework for Reporting Adaptations and Modifications to Evidence-based interventions (FRAME) [18, 20, 51] encompasses eight elements: (1) when and how the modification was made, e.g. pre-implementation, at scale-up, or during sustainment; (2) whether the modification was planned (proactive) or unplanned (reactive); (3) who decided on the modification, e.g. program leaders, funder, administrator, or recipient; (4) what was modified, such as content, context, training and evaluation, or implementation activities; (5) delivery level of the modification, e.g. individual or group, clinic level, organization, or community; (6) the nature of the context or content modification, such as tweaking, adding elements, loosening the structure, integrating the intervention into other frameworks or protocols, or drifting from the intervention protocol; (7) fidelity-consistency, meaning whether core elements and functions were preserved or changed; (8) the reasons for the modification, such as the goal of the modification, e.g. increase engagement, retention, fit, or to reduce cost, and reason behind the modification, e.g. sociopolitical, organizational, provider-specific, or recipient-specific. The eight elements have been presented in their entirety by Stirman et al. [20].

### Framework for analyzing determinants of implementing the intervention: CFIR

The Consolidated Framework for Implementation Science (CFIR) [17, 47] is an implementation determinant framework encompassing 48 distinct constructs across five domains. The five domains focus on the innovation itself, that is the intervention, practice, or protocol being implemented, the Outer Setting, such as the state or system around the inner setting in which the implementation takes place. Further, the Inner Setting in which the implementation takes place, such as a facility or clinic, the individuals’ characteristics and roles, and finally, the Implementation Process, such as the implementation strategies used and the degree to which these are executed. CFIR can be utilized in a variety of ways, including as an implementation planning tool and for post-trial evaluation [47, 53–55].

## Results

**Fig. 2:**
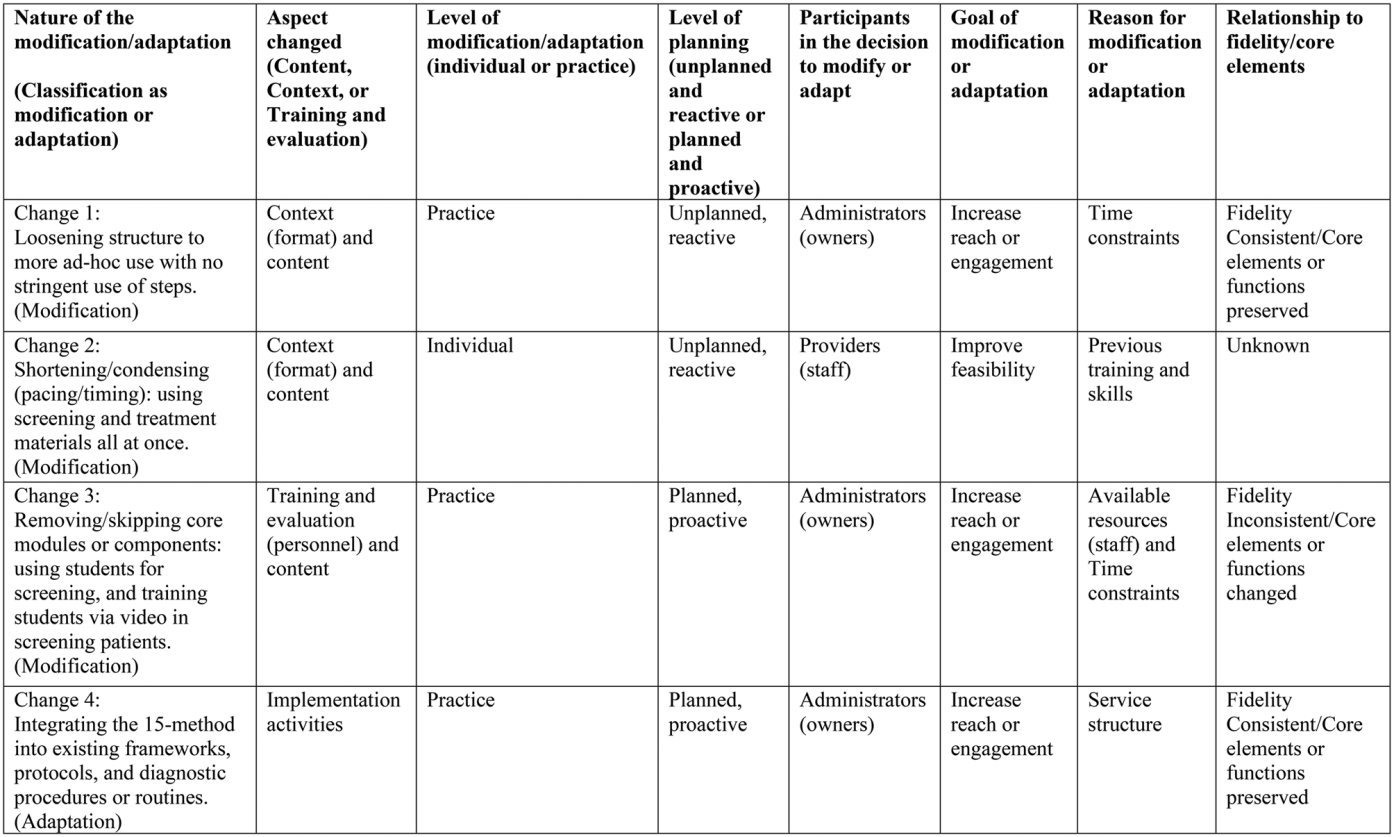
Notes: All modifications and adaptations occurred during implementation of the 15-method in the Identification and Treatment of Alcohol Problems in Primary Care (iTAPP) Study. Each modification and adaptation occurred in a separate practice. Table based on the Framework for Reporting Adaptations and Modifications to Evidence-based interventions (FRAME) by Stirman et al. 2019

We identified three unique modifications, with each modification occurring in a separate practice, as well as one adaptation observed in a fourth practice. Figure 2 provides an overview of these four changes, categorized according to the FRAME.

### Change 1: Loosened structure (modification)

The first modification involved adjusting both context and content of the 15-method, with a loosening of the method’s structure in favor of an ad-hoc use of the method’s materials rather than strictly following the method’s original structured steps. This unplanned modification, initiated by the GPs, aimed at increasing patient reach and engagement. The GPs described how they were using the 15-method as a general mindset regarding alcohol awareness, rather than as a fixed protocol. One of the primary reasons for this modification was to accommodate patient-centered consultations when being short on time. The GPs who made this modification said that they wanted to leave room for the patient to raise any topic or concern, which could often make the consultation messy. Some GPs viewed the habit-change process as an ongoing journey, needing flexibility to address fluctuations in patient motivation and resources, and reasoned that they therefore needed a looser structure of the 15-method to make it applicable to time constrained consultations with shifting agendas. By using a loose structure and selectively offering patient materials, the GPs maintained an “open door” approach, allowing patient motivation to drive the process. This first modification retained core elements of the 15-method, such as opportunistic screening, MI, homework assignments, and follow-up, and thus was considered fidelity-consistent.

Examples quotes:

*I think of it this way; it (altering drinking habits) is a process*,* and we need to have an eye on our own time. This project (iTAPP) is one aspect of beginning this process. It might be that it is not as stringent*,* and only a few patients get a follow-up*,* but just the fact that we have an increased focus (on alcohol) will definitely help decrease consumption among our patients. (HCP 6)*

*I can use the general idea*,* the mindset of screening patients for dependence and the mindset of individual goal setting. As in*,* where do you want to go in the future*,* what do you want to achieve and how would you like to work on this*,* so your addiction is not holding you back. I try to see (the patients) several times*,* try to make a course of treatment - to have an open door. But it can be difficult to hand over the patient materials. I don’t want to be authoritarian saying now it’s time for homework assignments and this and that. I see the materials as something they can read at home. (HCP 28)*

### Change 2: Condensed use of materials (modification)

The second modification, primarily seen among nurses, involved making use of all the 15-method materials at once rather than using it stepwise during more than one consultation. This modification was unplanned and occurred when staff faced time constraints, struggled with remembering specific steps or materials, felt overwhelmed by the materials, or felt unprepared for further discussions on alcohol habits. The HCPs stated that their MI or communications training was limited, which left them feeling overwhelmed by the method’s options. This led them to hand out all materials simultaneously. The nature of this modification was thus a shortening or condensation of the method, as screening and treatment materials were used all at once. Whether this condensed use of the 15-method was fidelity-consistent is unclear. The 15-method materials are developed for guided self-change and can largely be used as bibliotherapy, under the right circumstances. However, the fact that the HCPs expressed that they did not feel adequately trained in MI and uncomfortable going further into a discussion on alcohol habits indicate a fidelity-inconsistency, as the 15-method is premised on an MI approach and encompasses both screening and subsequent exploration of motivation, patterns, and treatment.

The following quote illustrates how an HCP felt overwhelmed by the complexity of navigating a potentially sensitive topic when not feeling adequately comfortable in MI, and getting lost in the 15-method materials under the time pressure of a brief consultation with other topics to cover:

*… that might be it - that there is too much to choose from*,* and we end up doubting what to include… Ultimately*,* I just handed (the patient) the AUDIT and the logbook – I think. We have a scheduled appointment coming up*,* but I haven’t talked to her since. (HCP 12)*

### Change 3: Delegated screening procedures (modification)

The third modification regarded using staff not enrolled in the overall iTAPP study as patient contact points for screening. Using different staff groups and assistants for lifestyle habit screening, routine examinations, or blood sampling is common in Danish general practice. However, to make up for time constraints, long waiting lists, and low staff resources, some GPs mobilized other staff groups, such as medical students assisting in the practice, to screen for alcohol problems in relation to the iTAPP project, without 15-method training. The stated intention behind this was to be able to refer a patient “inhouse” to an HCP trained in the 15-method if needed following screening. This modification was planned by the GPs, who utilized the opportunity to screen many patients through the student consultations, and instructed the medical students to hand out the AUDIT questionnaire to patients exceeding national alcohol consumption recommendations (maximum 10 standard drinks per week, and a maximum of four standard drink in one day), and schedule a follow-up with one of the staff members trained in the 15-method if needed:

*I have tried to figure out how we can use it (the 15-method) some more. I have recorded a video to our medical students who are the ones doing our blood sampling - and they are never here at the same time. So*,* I made video which I uploaded to their Messenger group chat where I talk about the 15-method and show them the AUDIT and tell them to hand out the AUDIT if they have a patient who drinks more than 10 drinks per day. I also tell them to ask the patient empathically about alcohol habits and whether the patient would be interested in participating in a project. I haven’t received any responses from patients. (HCP 26)*

We classified this modification as fidelity inconsistent as the students were not trained in the 15-method and had no knowledge on nor access to additional materials beyond the AUDIT and had no MI training.

### Change 4: Integration into existing procedures (adaptation)

The fourth change was classified as an adaptation and was a planned integration of the 15-method into existing clinical procedures in the practices. Although the 15-method training of HCPs the iTAPP study includes suggestions for implementation of the method into established workflows, neither the implementation strategy nor the method itself include any specifics as to where or when the method should be integrated. The intention behind this was to achieve maximum flexibility and to enable the HCPs to include the 15-method wherever they found it most useful and suitable. The practices most frequently integrated the 15-method into routine appointments such as controls for hypertension, weight, or diabetes. Additionally, some HCPs added the 15-method to existing diagnostic procedures in the practice, such as their diagnostic algorithm for sleep difficulties, which often includes questionnaires and monitoring sleep and lifestyle in a diary. This adaptation was made at the practice level, involving both GPs and nurses. We classified this adaptation as fidelity consistent, as the HCPs maintained the 15-method’s core elements and MI approach.

The following two examples illustrate how the 15-method was integrated into different types of consultations and existing clinical procedures in two different practices:

*When we assess sleep problems for instance*,* there is a lot of registration and diary work. It helps clarify the connection between good and bad habits. It (the 15-method) has been a part of this. Alcohol is one of the things the patient notes. (HCP 27)*

*I think the easiest time to talk about alcohol is when the patients want medicine for obesity. It’s easy because alcohol is a source of so many calories and we talk about what they eat and drink*,* their habits and so on. So*,* I schedule a follow-up and ask them how many beers they would normally have for lunch and whether they need any help from me (reducing consumption). (HCP 18)*

## Discussion

We identified four changes to the 15-method during its implementation in a randomized controlled trial in Danish general practice. Three were modifications, defined here as reactive changes in response to constraints or unforeseen challenges, and consisted of loosening the method’s structure, a condensed use of materials, and delegated screening procedures. The fourth change was an adaptation, i.e., an intentional and planned change with a focus on maintaining fidelity and was an integration of the 15-method into existing clinical procedures. Overall, the stated goal for these changes was most often to increase patient reach and engagement, but HCPs also reasoned that they made modifications because they were pressed for time or found the method complex to navigate. The latter was especially evident in cases where HCPs felt insufficiently trained in MI, a core aspect of the 15-method’s approach.

The adaptation and two out of three modifications were carried out by the administrators (GPs) and occurred on practice level. The adaptation of integrating the 15-method into existing routines was classified as fidelity consistent, and the method seemed to integrate well into existing procedures in the practices when staff felt comfortable using the method. Contrary, the un-planned modification of condensing the materials into one single hand-out was the only change carried out on provider (staff) level and was described as a reaction to feeling uneasy in both navigating the 15-method and the MI approach when being pressed for time. This suggests that these HCPs were inadequately trained in the 15-method or that implementation in the practices may have lacked structured feedback and progress monitoring to address such challenges among participants [16, 56, 57]. These findings align with our parallel analysis of individual-level implementation factors, which demonstrated that HCP confidence, self-efficacy, and perceived opportunities to deliver alcohol interventions significantly influenced fidelity to the 15-method [45].

It is possible that because this subset of HCPs felt insecure using the 15-method, they were unable to integrate it fluently into their work habits, causing them to spend excessive resources navigating the method’s options [58, 59], resulting in overwhelm and low-fidelity use of the method. Our parallel study examining individual characteristics further revealed that HCPs with lower baseline confidence in addressing alcohol issues were more likely to modify intervention components, corroborating the present findings regarding material condensation and structural modifications [45]. These findings features good examples of common challenges in implementation efforts, such as the tension between acceptability and appropriateness, and the effect of modifications on fidelity, adoption, and sustainability [22, 60, 61]. Regarding acceptability and appropriateness, the HCPs generally found the 15-method to be appropriate, that is, relevant, compatible, and useful to their work [61, 62]. However, our findings also indicate that the perceived acceptability of the method varied, i.e., the satisfaction with aspects of the method such as its complexity and comfort during delivery [61], most clearly illustrated in the cases of condensed use of the method’s materials and loosening of its structure. In other words, the HCPs generally found the method relevant, but to be comfortable delivering the method, and to do that in a fidelity-consistent way, a subset of HCPs needed more training and/or additional support tailored to individual competency levels. This can be addressed through e.g., practice specific pre-intervention skills and needs assessment, with tailored training and follow-up sessions to address variations in contexts and resources across practices [45].

Regarding adoption and sustainability, the modification of loosening the 15-method’s structure was un-planned, but fidelity-consistent, while the planned modification of delegating screening procedures to accommodate resource constraints was considered fidelity-inconsistent. When adapting or modifying an intervention, whether it is planned or not, it can generally entail the intervention’s “core components” or “adaptable periphery” [63]. Core components are essential to the intervention, and if altered, the change will most likely be fidelity-inconsistent. However, fidelity is in itself not a valuable measure, as a highly adaptable intervention may facilitate better adoption—i.e. the utilization and initial implementation of the intervention—and ultimately higher sustainability [61, 62]. Sustainability can be thought of as the continuation, incorporation, or integration of the intervention and is an indicator of later-stage implementation success [22, 61].

Developing and adjusting health interventions in complex systems, like the primary care sector, is not linear but an iterative process, requiring a back and forth between theory and practice [24] and changes to the intervention are bound to occur in different contexts [23]. Changes may be anticipated but are just as often emergent or opportunity-based [64]. The loosening of the method’s structure could be categorized as an emergent modification, that is, un-anticipated, un-planned, and spontaneously arising in a local context. The planned integration of the 15-method into existing procedures may, on the other hand, be categorized as an opportunity-based adaptation, purposefully introduced in response to the opportunity being presented, in this instance the introduction of the 15-method as an option within the practice. Adaptations and modifications can generally occur during a certain “window of opportunity”, which, when closed, leaves little room for further changes as the intervention gradually becomes discarded if not adequately embedded in everyday use, or becomes an engrained routine, sometimes even with unresolved problems or insufficient use of resources [65]. This entails that a re-opening or prolonging of the “window” may be necessary if part of the intervention or its utilization is considered sub-optimal [66]. Prior to the iTAPP study, the 15-method was adapted with a focus on its “adaptable periphery” to improve its fit in Danish general practice [14], while maintaining core components such as its MI approach and guided self-change framework. Our present findings indicate that the 15-method’s core elements are broadly accepted by HCPs, and the method is implementable, but its acceptability, and thus ultimately its sustainability and effectiveness, could be increased by prolonging the open window of opportunity and paying special attention to future implementation processes around the method. This includes revising its training sessions with a stronger focus on making HCPs comfortable using MI, planning for a looser application of the method’s structure, and providing closer follow-up and feedback to HCPs. Additionally, selecting implementation strategies that accommodate ongoing, context-driven changes to intervention components while maintaining fidelity to core components and can respond to emerging implementation challenges and opportunities.

Brief alcohol interventions are strongly supported for their effectiveness [4], but the last four decades of research show that universal screening of patients and strict SBI protocols are challenging to sustain in routine healthcare [10]. To advance SBI research, the implementation and adaptability of these brief interventions must be areas of focus, considering the large variation in healthcare contexts and stagnant uptake of alcohol interventions. In this regard, the present study exemplifies both a context-sensitive intervention that appears capable of accommodating differences in healthcare contexts, and that real-world changes to such an intervention can be readily incorporated into future iterations and related implementation strategies.

### Methodological considerations

First, we collected data during the initial implementation stage and approximately halfway through the iTAPP study. This entails that modifications in later stages of implementation were not considered. It is likely that modifications occurred later as routines would settle in, and the longer time horizon would imply a higher probability of unforeseen contextual factors affecting utilization of the method [23]. However, multiple time point assessments, in this case two, do provide a better understanding of how and why modifications occurred as changes can be examined over time, and provide more detail as to how the modifications could affect intervention sustainability [20, 22].

Second, additional data sources, such as observations or self-reports, would likely provide greater detail to the understanding of reasons for the modifications and can in some instances help shed light on unique patterns not easily identified from a single data source [67].

Third, the interviewer (PNS) is part of the iTAPP project group and part of the 15-method trainer team. This raises a potential risk of biases, especially confirmation bias and observer bias, along with potential conflicts of interest [68]. To mitigate these risks, we ensured structured group discussions focusing specifically on potential biases and collaborated with an external qualitative specialist to oversee analysis. Oppositely, the detailed knowledge on the 15-method and the implementation context, along with frequent contact to the participating HCPs, realized through an inside evaluator, allowed for nuanced insights to the implementation process and the recognition of subtle changes or modifications to the intervention [69].

Finally, our analysis focused on determinants, including the implementation process and related activities, from the perspective of HCPs. This approach introduces a risk of self-report and social desirability bias [68] and excludes insights from the iTAPP research team regarding the implementation process. A separate paper evaluating the overall iTAPP study process is planned.

## Conclusion

The 15-method can be considered acceptable for use by healthcare professionals in general practice and seems possible to implement with few adaptations. More training in the method and a strong focus on implementation planning may increase healthcare professionals’ comfort in using the method and help increase its sustainability. Forthcoming estimates of the 15-method’s effectiveness in real-world settings will help decide on and guide future larger scale implementation strategies.

## Supplementary Information

Below is the link to the electronic supplementary material.

Supplementary Material 1

## Data Availability

No datasets were generated or analysed during the current study.

## Abbreviations

SBI: Screening and Brief Intervention
HCP: Healthcare Professionals
iTAPP: Identification and Treatment of Alcohol Problems in Primary Care
CFIR: Consolidated Framework for Implementation Research
FRAME: Framework for Reporting Adaptations and Modifications to Evidence-based interventions
SRQR: Standards for Reporting Qualitative Research
GP: General Practitioner
AUDIT: Alcohol Use Disorder Identification Test
ICD: International Classification of Diseases
MI: Motivational Interviewing
CBT: Cognitive Behavioral Therapy
OPEN: Odense Patient data Explorative Network
FAST: Fidelity, Adaptation, Sustainability, and Training
