# Supplementary material for: Adaptations and modifications to the 15-method in Danish general practice classified using the framework for reporting adaptations and modifications to evidence-based interventions (FRAME)
Source: Addict Sci Clin Pract. 2025 Oct 27;20:87. doi: 10.1186/s13722-025-00613-7 (PMC12557837; doi:10.1186/s13722-025-00613-7)
Supplement: Supplementary file 1 — Supplementary Material 1 [file 13722_2025_613_MOESM1_ESM.docx]

*Not all questions were presented in every interview. Lead questions (prioritized questions) are highlighted in bold while remaining questions under the same heading were used as follow-up questions or probes. The guide is structured from the Consolidated Framework for Implementation Science interview guide resource available at www.cfirguide.org. This interview guide was used as part of a broader data collection strategy to address multiple research questions across related studies. For related publication focusing on domain IV and COM-B analysis see (Schøler, Sondergaard et al. 2025).*

**1. Background Information**

- What is your title and role in the clinic?
- How are you involved in the iTAPP project? (user of the 15-method, coordinator, supervisor, super-user)
- How was it decided that you would participate in the project?
- **How was it decided that the method would be implemented in this clinic?**

**2. Open description of the implementation**

- **I would like to hear about the process of implementing the 15-method in this clinic — in as much detail and with as many time points as possible: What are your thoughts and experiences with the method?**
- Has anything changed during the project?
- Who in the clinic uses the method?
- Who handles homework/reviews materials with patients?

**3. Domain-specific questions**

Now I would like to learn more about why you are participating and your general impression of the 15-method:

**(Innovation relative advantage Domain I)**

- **What do you think about using the method?**
- Does the method make it easier to ask about alcohol compared to what you used to do? If yes, how?
- To what extent does this intervention meet the actual need for an intervention in this area?

**(Tension for change Domain III)**

- **Has the method changed anything about how you discuss alcohol?** If something was particularly challenging before, has it become easier?

**(Innovation Evidence-base Domain I)**

- The 15-method has been tested abroad (Sweden), and we are testing its effectiveness here in Denmark. How significant is this for you?

**(Innovation Evidence-base Domain I; Innovation Design Domain I)**

- What do you think of the 15-method overall?
- What do you think about its credibility?

**(Complexity Domain I)**

- **Is it (the 15-method) easy or complex to use?**
- What do you think about the structure of the method (the steps)?
- Do you have the necessary overview?
- How does the material align with the idea/concept behind the method? (a stepwise flexible approach)

**(Innovation Design Domain I)**

- What do you think is good/less good about the quality, design, and layout of:
  - The manual?
  - The overview card?
  - The logbook?
  - The flyers?
  - The AUDIT form?

**(Relative Priority Domain III and Available Resources Domain III)**

- Do you have any other new/large initiatives in the clinic that are being implemented alongside this intervention?
- **How does this intervention/the use of the method rank compared to other activities in the clinic — is there time and capacity to use it?**
- Does the 15-method take time away from other tasks?
- Are these other projects/priorities short-term or long-term?

Now I would like to hear more about your experiences with implementing the 15-method in this clinic.
There are no right or wrong answers, and there is no single way to do it. I would also like to hear more about any changes you may have made or considerations/desires for changes you may have.

**(Access to Knowledge and Information Domain III)**

- Do you have access to the material/information you need to use the method?
- What is missing, if anything?
- **Have you received sufficient information and training in the method to start using it (the 15-method)?**
- Is there a need for case examples and training before it is put into use?
- Is there sufficient communication with the project team for support?

**(Planning Domain V)**

- How did you plan to implement the method in your daily work?
- Can you describe the plan?
- Who did what?
- If there is a plan, do you have any milestones?
- How are you progressing toward these goals?

**(Compatibility Domain III)**

- **How does the method fit into your normal workflows?**
- Does it make any processes or types of work easier/harder and why?

**(Adaptability, modifications)**

- **Which professional groups in the clinic use the method, and how does it work?**
- How do you collaborate on this?
- What is the role of the doctors compared to the nurses?
- Is there support from the leadership for the other staff?
- If only participating nurses: Are the doctors aware of the project, and are there disadvantages/advantages in this?
- Was anyone specifically designated, or did people volunteer?
- Has there been a shift in who uses the method the most compared to when you started?
- How do you adapt the method – if so, how?
- Is the method flexible enough to fit into your daily routine? Why/why not?
- How could it better account for unique differences in the clinic?
- **Are you actively doing something to adapt it?**
- Have you made any physical or other changes to your environment to use the method?
- Any other changes or modifications you can think of?

**(Communications Domain III)**

- **How do you communicate about the project and the method — formally (meetings, emails) or informally (in the hallway, during breaks)?**
- What has been the most useful?
- Would you like more of the other (formal/informal)?
- Can you give examples related to the 15-method?

**(Work Infrastructure Domain III)**

- **How do you divide the tasks related to the 15-method among you?**
- Have you specified roles/responsibilities?

**(Complexity Domain I + Doing Domain V)**

- On a scale from 0 to 10, where 0 is the easiest thing in the world and 10 is impossible, how difficult would you say it was/is to implement the 15-method in your clinic?
- Were there specific bottlenecks?
- Have there been any unforeseen demands for time or other challenges that have consumed more resources than expected?

**(Doing Domain V)**

If the method has been implemented:

- From your own perspective, on a scale from 0 to 10, where 0 is a failure and 10 is a success, how successful do you think the 15-method is in this clinic after it has been put into use?
- What do you base this assessment on?
  *NOTE: Focus here is on what participants perceive as a success.*
- **What has been good/less good in terms of getting the method used in the clinic?**
- Have there been any major incidents or unforeseen changes/limitations that have affected the implementation or use? (critical incidents, Domain II)
- Are there any local factors that make it easier/harder to use the method (other initiatives in the municipality, collaboration with local institutions, socio-cultural aspects)?

**(Reflecting and evaluation Domain V)**

- **How (if at all) do you follow up on the implementation of the method in the clinic?**
- Have you brought it up in monthly/weekly meetings?
- Have you allocated time specifically for reflection or evaluation?

**(Doing Domain V; available resources Domain III)**

- **What is necessary to ensure that a new method continues to be used (sustained use)?**
  ... In general practice?
- Do you feel that the necessary resources have been accounted for in this project?

**(Innovation Cost Domain I)**

- What role does economics play?

**(Partnership and Connections Domain II)**

- How do you assess your collaboration with organizations/institutions outside the clinic in this area? (referrals, municipal services)

**(Capability Domain IV (sub-domain Characteristics), COM-B system)**

- **Do you feel that you have the skills and knowledge to use the method?**
- ... Skills to use the method the way you would like to?

**(Opportunity Domain V (sub-domain characteristics), COM-B system)**

- Do you feel that you have opportunities to change things in the clinic if you set your mind to it?
  For example, activities to strengthen skills (preferably related to the 15-method)?
- Opportunities to change conditions related to the 15-method (structure, workflows, material use)?

**(Motivation Domain V (sub-domain characteristics), COM-B system)**

- How important is the issue of alcohol in your practice?
- How ready are you to work on this issue?
- **What drives your engagement in working with the method?**
- Do you feel motivated in this area (alcohol)?

**(Innovation Recipients Domain IV (sub-domain Roles); Need Domain IV (sub-domain Characteristics); Recipient Centeredness Domain III culture)**

- **What do you think the patients think about the method?**
- Do you have examples of specific experiences, conversations, or situations with patients?
- Do you have examples of specific expectations from the patients regarding the method?
- Do patients find it difficult/easy to follow the material/progression of the method?
- Has age been a factor in the use/engagement with the method?
- Has gender been a factor?
- Have you experienced patients refusing to use the tools in the method? If so, do you know what led them to refuse?
- Are there elements that appeal to certain patients/situations?
- Are there parts that work particularly well for certain patients/consultations?

**Finally, considerations and suggestions for improving the method:**

- **Do you have any ideas or considerations for improving the 15-method itself?**
  The material (Manual, Logbook, Flyers, Overview card, AUDIT form)?
  The structure (steps)?
  Tips and tricks section?
  Supporting quotes?
  Digital options?
- Do you have any reflections on the introduction, training, and getting the method started in the clinic?
  Support, feedback?
  Assistance in getting the method incorporated into daily work/implementation support?
- Anything else you would like to comment on or have thoughts about?

Reference:

Schøler, P. N., J. Sondergaard, S. Rasmussen, K. H. Volke, P. Nilsen and A. S. Nielsen (2025). "Determinants of implementing the 15-method in Danish general practice using the consolidated framework for implementation science." Addict Sci Clin Pract **20**(1): 43.
